# Non-invasive Prefrontal/Frontal Brain Stimulation Is Not Effective in Modulating Food Reappraisal Abilities or Calorie Consumption in Obese Females

**DOI:** 10.3389/fnins.2017.00334

**Published:** 2017-06-20

**Authors:** Felicitas Grundeis, Cristin Brand, Saurabh Kumar, Michael Rullmann, Jan Mehnert, Burkhard Pleger

**Affiliations:** ^1^Department of Neurology, Max Planck Institute for Human Cognitive and Brain SciencesLeipzig, Germany; ^2^Collaborative Research Centre 1052 “Obesity Mechanisms”, University Hospital LeipzigLeipzig, Germany; ^3^Department of Systems Neuroscience, University Medical Center Hamburg-EppendorfHamburg, Germany; ^4^Department of Neurology, BG University Clinic Bergmannsheil, Ruhr-University BochumBochum, Germany

**Keywords:** obesity, non-invasive brain stimulation, transcranial direct current stimulation, dorsolateral prefrontal cortex, frontal operculum, reappraisal of food, eating, calorie consumption

## Abstract

**Background/Objectives:** Previous studies suggest that non-invasive transcranial direct current stimulation (tDCS) applied to the prefrontal cortex modulates food choices and calorie intake in obese humans.

**Participants/Methods:** In the present fully randomized, placebo-controlled, within-subject and double-blinded study, we applied single sessions of anodal, cathodal, and sham tDCS to the left dorsolateral prefrontal cortex (DLPFC) and contralateral frontal operculum in 25 hungry obese women and investigated possible influences on food reappraisal abilities as well as calorie intake. We hypothesized that tDCS, (i) improves the ability to regulate the desire for visually presented foods and, (ii) reduces their consumption.

**Results:** We could not confirm an effect of anodal or cathodal tDCS, neither on the ability to modulate the desire for visually presented foods, nor on calorie consumption.

**Conclusions:** The present findings do not support the notion of prefrontal/frontal tDCS as a promising treatment option for obesity.

## Introduction

The rapid worldwide spread of obesity and associated comorbidities such as diabetes, cardiovascular diseases, and cancer (Dixon, 2010) as well as its complex etiology and inter-individual variability in response to intervention demand the development of new therapeutic strategies (Roman et al., 2015). The majority of currently available weight loss programs are based on dieting and physical activity (Jakicic and Davis, 2011; Amorim Adegboye et al., 2013; Soeliman and Azadbakht, 2014), which on the one hand are accessible and affordable, but on the other hand often lead to timely restricted effects followed by rapid weight regain after the program has ended (known as the yo-yo effect).

Bariatric surgery (weight loss surgery) is currently seen as the gold standard in the therapy of obesity, since it proved to be effective in inducing lasting weight loss (Sjöström, 2000; Buchwald and Oien, 2013). However, high costs and surgery-associated risks leave it to be an exceptional option for only morbidly obese individuals. Since most weight-loss programs approach obesity on the symptomatic level, the treatment of underlying causes, repeatedly shown to be brain-dependent, appear indispensable for developing new therapeutic strategies leading to lasting weight loss and healthier living.

Converging evidences agree on the notion that obesity affects the structure (Hollmann et al., 2012; Cone et al., 2014) and function of the central nervous system (Hollmann et al., 2012) in relation to dysregulated hormonal feedback from the digestive system (Schlögl et al., 2016). Whereas, homeostatic control sites in the hypothalamus integrate and process information from the body's periphery to ensure energy balance (Morton et al., 2006), food cues such as sight, smell and taste affect hedonic brain regions involved in goal-directed and habitual behavior, such as the ventral and dorsal striatum, respectively (Wang et al., 2001; Saper et al., 2002; Stoeckel et al., 2008). The obesity-related, dysregulated feedback from the digestive system to those homeostatic and hedonic brain sites as well as the attenuated reward responsivity to food intake (Stice et al., 2008a) seem to maintain overeating behavior.

Especially high-caloric foods seem to affect the brain's reward responses like drugs of abuse (Volkow et al., 2013). Like drug addicts, obese individuals present increased craving as well as attenuated reward responses to high-calorie foods, probably supporting compensatory overeating (Wang et al., 2001; Stice et al., 2008b; Johnson and Kenny, 2010). Dopamine seems to have a central role in mediating these effects. In obese rats, for instance, electrically evoked dopamine release in slice preparations was significantly attenuated, not only in the nucleus accumbens but also in additional terminal sites of dopamine neurons such as the accumbens shell, dorsal striatum, and medial prefrontal cortex, suggesting that there may be a widespread dysfunction in mechanisms regulating dopamine release in obesity (Geiger et al., 2008, 2009; Zhang et al., 2015).

However, the interplay between the hypothalamus and reward-related regions alone cannot explain the complex neurobiological mechanisms involved in food choices, such as those underpinning the appraisal or reappraisal of healthy and unhealthy food. Associated brain mechanisms are of potential interest for the development of new therapeutic strategies, such as neurofeedback training or non-invasive brain stimulation. In previous studies, we aimed to identify brain regions involved in those processes with functional magnetic resonance imaging (fMRI; Hollmann et al., 2012) and electroencephalography (EEG; Kumar et al., 2016). Findings suggest that an active reappraisal of tasty but unhealthy food recruits the brains valuation system in combination with cognitive control areas, such as the dorsolateral prefrontal cortex (i.e., DLPFC), and gustatory areas such as the frontal operculum, which together with the neighboring anterior insular cortex is assumed to host primary gustatory processes, such as taste perception (Rolls et al., 1988; Zatorre et al., 1992; Small et al., 1999). The DLPFC's activity increased during admitting the desire for high and low calorie food (Kumar et al., 2016), supporting the notion of the DLPFC's decisive influence on self-control (Hare et al., 2009) and cognitive reappraisal (Kober et al., 2010). The right frontal operculum's activity increased when regulating food desire, assigning higher cognitive functions, such as food imagery, to the primary gustatory cortex (Kumar et al., 2016). Based on these findings, neurofeedback training, or non-invasive brain stimulation based on the DLPFC's and frontal operculum's state-dependent activation levels could strengthen executive top-down control on food choices and food-related reward processing through modulating the DLPFC's and frontal operculum's functional implementation in an individualized manner.

In the present study, we investigated the role of the DLPFC and the frontal operculum in the active reappraisal of high or low calorie food, as well as their consumption using non-invasive transcranial direct current brain stimulation (tDCS). TDCS is a secure procedure for subliminal, tonic electric stimulation of the brain (Nitsche and Paulus, 2000; Brunoni et al., 2011). A weak direct current of 1–2 mA is applied to generate regional changes in cortical excitability, which, depending on the duration and the polarity, can last for several minutes up to a few hours after stimulation (Nitsche and Paulus, 2001; Hummel and Cohen, 2005). Whereas the neuronal effects during tDCS are characterized by a shift of membrane potentials in cortical neurons that lead to a modification in the regional neuronal activity, sustainable effects (i.e., the following 20 min after application) seem to be mediated by changes in the efficiency of synaptic transmission (Clark et al., 2011; Rahman et al., 2013). Studies in animals as well as humans indicate that anodal stimulation leads to an increase in neuronal excitability, whereas cathodal tDCS leads to hyperpolarization of the membranes and therefore causes decrease in neuronal excitability (Paulus, 2004; Been et al., 2007). However, this clear dichotomy seems to describe the effects in the motor cortex, which cannot be transferred to cognitive tasks *per-se* (Boehringer et al., 2013; Macher et al., 2014; Taubert et al., 2016). Previous studies investigating the effect of tDCS on the DLPFC suggest that anodal stimulation in lean individuals reduces food craving (Goldman et al., 2011; Montenegro et al., 2012; Kekic et al., 2014; Ljubisavljevic et al., 2016) and caloric intake (Fregni et al., 2008; Jauch-Chara et al., 2014) immediate to tDCS. The tDCS study by Gluck et al. appears to be the only study to date, probing repetitive application of tDCS to the DLPFC in an solely obese cohort, resulting in decreased daily kilocalories consumed and greater percentage of weight loss as compared to cathodal and sham (placebo) stimulation (Gluck et al., 2015).

Based on our recent EEG findings (Kumar et al., 2016) and the well-described dichotomic tDCS influences on the motor cortex (Paulus, 2004; Been et al., 2007), we here hypothesized, that tDCS, with the cathode placed over the left DLPFC, downregulates the DLPFC's activity, whereas the simultaneous anodal stimulation of the right frontal operculum simultaneously upregulates the frontal operculum's activity. The potential up- or downregulation in the two targeted areas (left DLPFC, right operculum), whose influence on food desire modulation in obese was evinced in our previous study (Kumar et al., 2016), could also trigger alterations of the obesity associated reduced dopamine response in central reward related regions such as ventral and dorsal striatum as well as nucleus accumbens (Geiger et al., 2008, 2009; Zhang et al., 2015) due to their tight interconnectedness. We expected that these tDCS influences strengthen the ability to regulate food desire and reduce calorie consumption as compared to the inverse tDCS polarity (i.e., anodal stimulation over left DLPFC, cathodal stimulation over right frontal operculum) as well as sham tDCS.

## Materials and methods

### Participants

Thirty-two healthy obese women were recruited by local and online advertisement of which 25, aged 18–43 (mean 28.8 ± 6 years) met the inclusion criteria for the study and completed all sessions (Table 1). BMI range was 31.4–45 kg/m^2^ (mean 36.5 ± 4.1 kg/m^2^) and all women were right-handed and naïve to non-invasive brain stimulation. Exclusion criteria implied (a) neurological and/or psychiatric illness, (b) depression (assessed by Beck's Depression Inventory, BDI index > 15; Hautzinger et al., 1994; Ivezaj et al., 2016), (c) smoking and/or drug abuse, (d) pregnancy (appraised by a urine rapid test at first session) and nursing, (e) current dieting and/or participation in weight loss programs, (f) diabetes and (g) contraindications for tDCS (such as metal implants, history of seizures, migraine, neurosurgery, or sleeping disorders). Menstrual cycle was not inquired. Of the initially recruited 32 women, two were excluded due to indication of major depression, two conducted a vegetarian diet, one had multiple food allergies, one reported a diagnosed elevated cortisol level with unclear cause and one did not return after her first session. Two of the 25 remaining participants submitted an insufficient amount of task self-ratings (missings > 50%), discovered during subsequent data analysis and have therefore been excluded solely from analyses of self-ratings. All volunteers provided written informed consent and were financially reimbursed for their participation. The study was approved by the ethics committee of the medical faculty of the University of Leipzig and conducted in accordance with the declaration of Helsinki.

**Table 1:** Mean, standard deviation, and range for age, weight, and BMI of participant cohort.

	**Age [years]**	**Weight [kg]**	**BMI [kg/m^2^]**
Mean	28.8	102.5	36.5
Standard deviation	6.0	11.8	4.1
Range	18–43	82–130	31.4–45

### Questionnaires and visual analog scales

**Figure 1:**
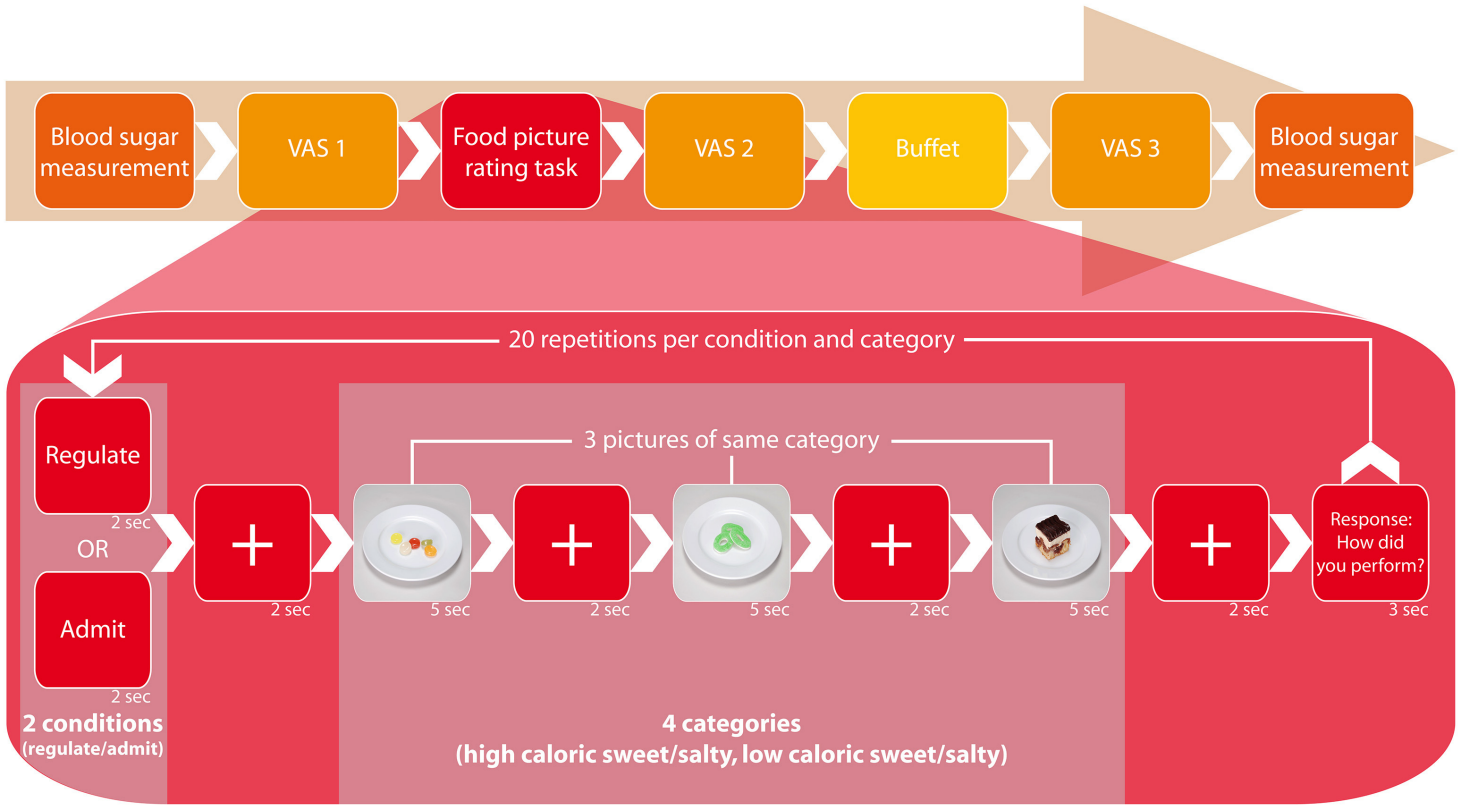
Study design. The figure displays the process of one session, which proceeded identically each session and independent from tDCS type. At the outset, we measured blood glucose levels from fingertip blood samples, followed by the first presentation of visual analog scales (VAS 1). Simultaneously to the ensuing food picture rating task, the participants received one of three tDCS types (anodal, cathodal, or sham) for 20 min in a randomized and double-blinded setting. During the task, they were visually instructed to either admit or regulate their desire for three displayed food pictures and rate their ability to follow that prompt on a range from 1 (“very good”) to 4 (“poorly”) thereafter. Food pictures were randomized in their display and associated to four categories: *high caloric sweet/salty* and *low caloric sweet/salty* (2-by-2 factorial design). One set of three pictures was associated with one category and each picture was presented twice: once for the regulate and once for the admit condition, summing up to a total of 20 performance ratings for each condition. After the experiment followed the second presentation of VAS (VAS 2). Thereafter we offered a standardized buffet containing the same food items as presented during the task. At the end of each session, participants completed the VAS a third time (VAS 3) and blood glucose levels were measured for comparison.

When coming to their first session, all participants were given a set of baseline assessments for screening purposes and to explore personal traits, which have a relevant impact on eating behavior and cognitive control. In particular, participants completed the Beck's Depression Inventory (BDI) as instrument for depression screening (Hautzinger et al., 1994; Ivezaj et al., 2016), as well as the Eating Disorder Examination-Questionnaire (EDE-Q; Fairburn and Beglin, 1994; Black and Wilson, 1996; Hilbert et al., 2007), the Three Factor Eating Questionnaire (FEV; Stunkard and Messick, 1985; Pudel and Westenhöfer, 1989), the Barrat Impulsiveness Scale (BIS-15; Meule et al., 2011), the Impact of Weight on Quality of Life questionnaire, 31-item short form (IWQOL-Lite; Kolotkin et al., 2001; Mueller A. et al., 2011) and the Adult Temperament Questionnaire (ATQ; Wiltink et al., 2006) for verification that participants are representative for an obese female cohort. Besides, we asked the participants to answer six questions to assess possible changes in vegetative functions that might have been affected by staying without food for 5 h or eating to satiety: How tired are you?, How dry does your mouth feel?, How sated are you?, How stressed do you feel?, How hungry are you?, How thirsty are you? For each of the six questions we provided a Visual Analog Scale (VAS) ranging from 0 (not at all) to 100 (very). The line of each VAS was 100 mm long. The distance between 0 and the cross made by the participant in mm was applied to further analyses. The VAS were surveyed before the food picture rating task (pre-task, VAS 1), immediately after the food picture rating task (post-task, VAS 2) and after eating at the buffet (post-buffet, VAS 3; Figure 1).

### Transcranial direct current stimulation (tDCS)

The experimenter instructing the participants (FG) was blinded and unaware of the type of tDCS application until the end of the study. Another experimenter (CB, SK) attached the tDCS electrodes and monitored the stimulation. We delivered tDCS with 2 mA through a pair of surface-soaked sponge electrodes (7 × 5 cm) using a commercial tDCS device (NeuroConn, Ilmenau, Germany). Anodal and cathodal tDCS application lasted for 20 min including a 30 s fade-in and 30 s fade-out phase with a constant current phase of 19 min in between. Sham tDCS consisted of fade-in and fade-out only, thus avoiding actual stimulation while participants felt the initial tingling sensations associated with active current. This is an established placebo (or sham) condition (Fertonani et al., 2015). Anode and cathode were placed according to our recent findings of higher EEG activation in the left DLPFC while allowing the desire for food, and higher EEG activation in the right frontal operculum while regulating the desire for food (Kumar et al., 2016). Projecting these source locations on an averaged scalp surface using the look-up tables provided by Koessler et al. (2009) resulted in the EEG sensor localizations AF7 and F8 (10/10 system). We first transposed the MNI coordinates from Kumar et al. (2016) to Talairach space (Lacadie et al., 2008) and chose the closest coordinates (i.e., minimal Euclidean distance) for scalp EEG electrode positions. For “cathodal” stimulation, the cathode was placed over the left DLPFC (F8) and the anode over the right frontal operculum (AF7), respectively, and vice versa for “anodal” stimulation. Duration (20 min) and current density (0.057 mA/cm^2^) were chosen in line with determined stimulation protocols that are assumed to be effective whilst safe (Iyer et al., 2005; Nitsche et al., 2008) and provide comparability to previous studies in the field of interest (Fregni et al., 2008; Goldman et al., 2011; Montenegro et al., 2012; Kekic et al., 2014; Lapenta et al., 2014).

### Experimental design and study protocol

This study employed a fully randomized, sham-controlled, double-blinded, and within-subject crossover design. All participants received anodal, cathodal, and sham tDCS randomly assigned by a third party in three different sessions with an interval of at least 1 week to avoid carryover effects. Sessions occurred at the same daytime, 2–5 p.m., and after a fasting period of minimum 5 h (Kumar et al., 2016). The procedure of each session was identical (Figure 1). At the outset participants assessed the visual analog scales (VAS 1) and subsequently underwent 20 min of tDCS while simultaneously conducting a food picture task at a computer—the same task as in our recent EEG pilot that we conducted to identify the targets for tDCS (Kumar et al., 2016). During the task, participants were visually instructed to either admit or regulate their desire for displayed food pictures and rated their ability to following that prompt after a set of three pictures on a range from 1 (“very good”) to 4 (“poorly”). Food pictures were randomized in their display and associated to four categories: *high caloric sweet/salty* and *low caloric sweet/salty* (2-by-2 factorial design). One set of three pictures was associated with one category and each picture was presented twice: once for the regulate and once for the admit condition, summing up to a total of 20 self-ratings for each condition. Consequently, both, the admit and the regulate condition were implemented in each session, random in order but equally distributed. Directly after the experiment and tDCS application, participants were asked to reassess the VAS (VAS 2) before they were offered a standardized buffet containing the same food item as presented during the task (Figure 1). Arranged in a separate room, participants were told to eat to repletion *ad libitum*. All 20 food items were measured (in g) before and after the buffet with a standard kitchen scale to compute the consumed g per item. We used the kcal/100g indications provided on the products' packages to translate consumed g into kcal. For fruit/vegetables we took the brand-specific kcal/100 g indications as provided by the food database (fddb), accessible via http://fddb.info/db/en/index.html. The entire buffet contained an average of 18 296 kJ [4 370.2 kcal; *SD* = 343 kJ (82.5 kcal)]. At the end of each session we once again asked for evaluation of the VAS (VAS 3). Blood sugar levels were measured at the beginning and end of each session using the ACCU-CHECK Aviva blood glucose meter (Roche Diabetes Care, Mannheim, Germany) to analyze capillary blood from the fingertip (mmol/l; Table 2).

**Table 2:** Mean blood glucose levels before and after eating for anodal, cathodal, and sham tDCS.

	**Mean blood sugar pre-buffet ± standard deviation [mmol/l]**	**Mean blood sugar post-buffet ± standard deviation [mmol/l]**
**Anodal**	5.3 ± 0.6	6.6 ± 1.2
**Cathodal**	5.5 ± 0.7	7 ± 1.4
**Sham**	5.4 ± 0.6	6.6 ± 0.8

### Data analysis

Statistical analyses were performed using IBM SPSS software (Ehningen, Germany). We used repeated measures of analysis of variance (RM-ANOVA) to investigate if there was an effect of condition (anodal vs. cathodal vs. sham) as independent variable on (1) the performance ratings (admit vs. regulate) and (2) the total caloric intake as dependent factors. Mauchly's Test of Sphericity was performed to test the assumption of sphericity. *Post-hoc* paired *t*-tests were used to further decipher the structure of significance (*p*-value of 0.05 indicated significance).

## Results

All women tolerated tDCS well. Reported side effects like headache, dizziness, or burning sensations were only temporary and did not lead to premature discontinuation of an experiment session.

### Questionnaires

Twenty participants achieved 0–8 points in the BDI, indicating no signs of depressive symptoms, three reached 9–13 (minimal depression) and two reached 14 or 15 (mild depression) points. All questionnaire scores acquired through the IWQOL-Lite, EDE-Q, BIS-15 and ATQ conduced to verifying the comparability to society cohorts and means all were found within representative ranges given by literature (Kolotkin and Crosby, 2002; Wiltink et al., 2006; Hilbert et al., 2007; Spinella, 2007; Table 3). Correlation of the three FEV subscales (*cognitive restraint of eating, disinhibition, hunger*) with BMI showed no significant relation. Former findings of Hilbert et al. (2007) evincing a strong relation of *cognitive restraint of eating* with the EDE-Q subscale *restraint* could be replicated (*r* = 0.49, *p* < 0.02, *n* = 25). Further correlations of the *cognitive restraint of eating* and *disinhibition* subscales with regulating performance ratings and caloric intake did not show the expected association. Correlation analysis of BMI and BIS-15 as well as ATQ, both representing parameters of impulse and temper controlled personal traits, did not confirm a verifiable coherence.

**Table 3:** Mean values of questionnaires and associated reference values.

**Questionnaires**	**Mean value ± standard deviation**	**Reference mean value ± standard deviation**
IWQOL-lite	52.8 ± 27.7	61.2 ± 21.5
EDEQ	1.6 ± 1.08	1.44 ± 1.22
BIS-15	30.4 ± 4.5	32.6 ± 6.9
ATQ—EC	4.5 ± 0.6	*4.6* ± 0.1^*^
FEV—cognitive control	8 ± 5	10.08 ± 4.7
FEV—disinhibition	7.6 ± 2.9	10.58 ± 3.35
FEV—feelings of hunger	6.4 ± 4.2	7.8 ± 3.37

* *Estimated from published figure. Impact of weight on quality of life (IWQOL-lite; Kolotkin and Crosby, 2002). Eating disorder examination questionnaire (EDE-Q; Hilbert et al., 2007). Barratt impulsiveness scale—short version (BIS-15; Spinella, 2007). Adult temperament questionnaire—subscale effortful control (ATQ-EC; Wiltink et al., 2006). German version of the Three Factor Eating Questionnaire (FEV). Listed questionnaire scores conduced to verifying the comparability to society cohorts and means all were found within representative ranges given by literature*.

### VAS

**Figure 2:**
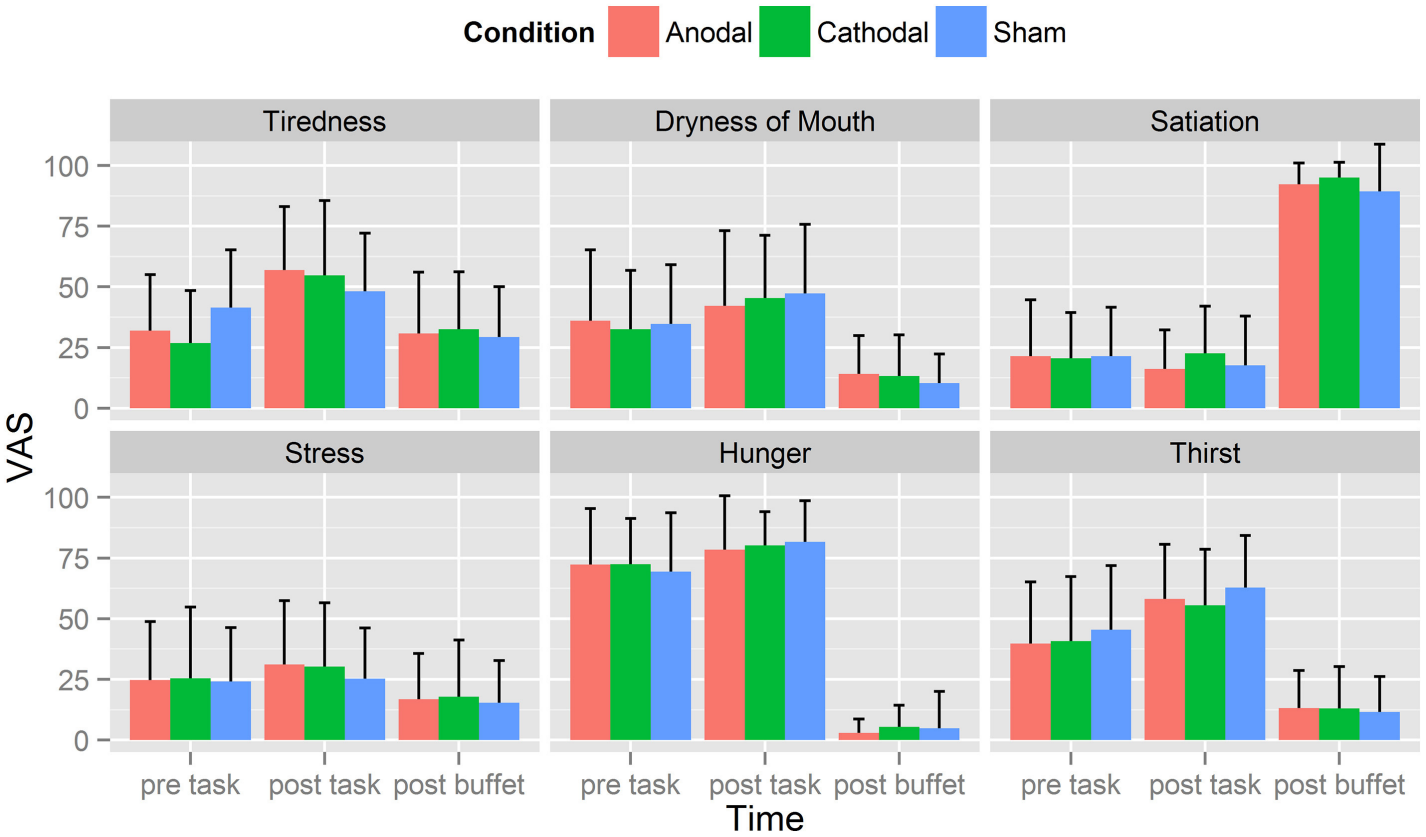
Visual analog Scales (VAS). Displayed are averaged self-ratings for the questions: How tired are you?, How dry does your mouth feel?, How sated are you?, How stressed do you feel?, How hungry are you? How thirsty are you? on a scale from 0 (“not at all”) to 100 (“very”). Each participant completed those questions three times: before the food picture rating task (pre-task), immediately after the food picture rating task (post-task) and after eating at the buffet (post-buffet). Participants were hungry at the outset of each session (*M* = 71.4, *SD* = 0.7) and moderately thirsty (*M* = 42.0, *SD* = 3). VAS values of tiredness, dryness of mouth, hunger and thirst significantly increased from VAS 1 to VAS 2 and decreased from VAS 2 to VAS 3. Values of satiation on the other hand decreased from VAS 1 to VAS 2 and increased from VAS 2 to VAS 3.

We assessed six VAS scales (0–100; tiredness, dryness of mouth, satiation, stress, hunger, and thirst) over three time points: 1st before tDCS (VAS 1), 2nd after tDCS (VAS 2) and 3rd after buffet (VAS 3; Tables 4A–C). Shapiro–Wilk test for normality revealed normal distribution of each rated category at given time points. Overall mean values showed that participants were hungry at the outset of each session (*M* = 71.4, *SD* = 0.7) and moderately thirsty (*M* = 42.0, *SD* = 3). VAS values of tiredness, dryness of mouth, hunger and thirst significantly increased from VAS 1 to VAS 2 (tiredness: *p* < 0.01, dryness of mouth: *p* < 0.01, stress: *p* = 0.04, hunger: *p* < 0.01, thirst: *p* < 0.01; *n* = 25) and decreased from VAS 2 to VAS 3 (tiredness: *p* < 0.01, dryness of mouth: *p* < 0.01, stress: *p* < 0.01, hunger: *p* < 0.01, thirst: *p* < 0.01; *n* = 25), whereas values of satiation decreased from VAS 1 to VAS 2 (*p* < 0.01, *n* = 25) and increased from VAS 2 to VAS 3 (*p* < 0.01; *n* = 25), for means see Tables 4A–C. RM-ANOVA could not show any significant difference between sham, anodal or cathodal tDCS conditions: hunger [*F*_(2, 48)_ = 0.246, *p* = 0.78, *n* = 25], satiation [*F*_(2, 48)_ = 1.291, *p* = 0.28, *n* = 25], thirst [*F*_(2, 48)_ = 0.331, *p* = 0.72, *n* = 25], stress [*F*_(2, 48)_ = 0.212, *p* = 0.81, *n* = 25], tiredness [*F*_(2, 48)_ = 0.062, *p* = 0.94, *n* = 25], dryness of mouth [*F*_(2, 48)_ = 0.013, *p* = 0.99, *n* = 25; Figure 2].

**Table 4:** Visual Analog Scale (VAS).

	**Anodal**	**Cathodal**	**Sham**
**A**. Before the food picture rating task (pre-task, VAS 1)			
Tiredness	26.8 (21.7)	31.9 (23.1)	41.5 (23.7)
Dryness of mouth	32.5 (24.3)	36.1 (29.2)	34.8 (24.3)
Satiation	20.6 (18.8)	21.5 (23.1)	21.5 (20.1)
Stress	25.4 (29.4)	24.7 (24.1)	24.1 (22.2)
Hunger	72.5 (18.8)	72.3 (23.1)	69.4 (24.3)
Thirst	40.8 (26.5)	39.8 (25.4)	45.4 (26.5)
**B**. After the food picture rating task (post-task, VAS 2)			
Tiredness	54.7 (30.8)	56.9 (26.1)	48.1 (24)
Dryness of mouth	45.4 (25.9)	42.2 (31)	47.3 (28.4)
Satiation	22.6 (19.4)	16.2 (16.1)	17.7 (20.2)
Stress	30.3 (26.3)	31.1 (26.4)	25.3 (20.9)
Hunger	80.2 (13.9)	78.5 (22.1)	81.7 (16.9)
Thirst	55.5 (23.1)	58.2 (22.4)	62.9 (21.4)
**C**. After *ad libitum* eating at the buffet (post-buffet, VAS 3)			
Tiredness	32.6 (23.6)	30.8 (25.2)	29.3 (20.7)
Dryness of mouth	13.2 (17)	14.2 (15.7)	10.4 (11.9)
Satiation	95 (6.3)	92.3 (8.7)	89.4 (19.4)
Stress	17.8 (23.4)	16.8 (18.8)	15.4 (17.3)
Hunger	5.5 (8.9)	3 (5.7)	4.8 (15.2)
Thirst	13 (17.2)	13.2 (15.5)	11.6 (14.5)

*Mean values and standard deviation for the questions: How tired are you?, How dry does your mouth feel?, How sated are you?, How stressed do you feel?, How hungry are you?, How thirsty are you? on a scale from 0 (“not at all”) to 100 (“very”)*.

### Self-rated task performance

**Figure 3:**
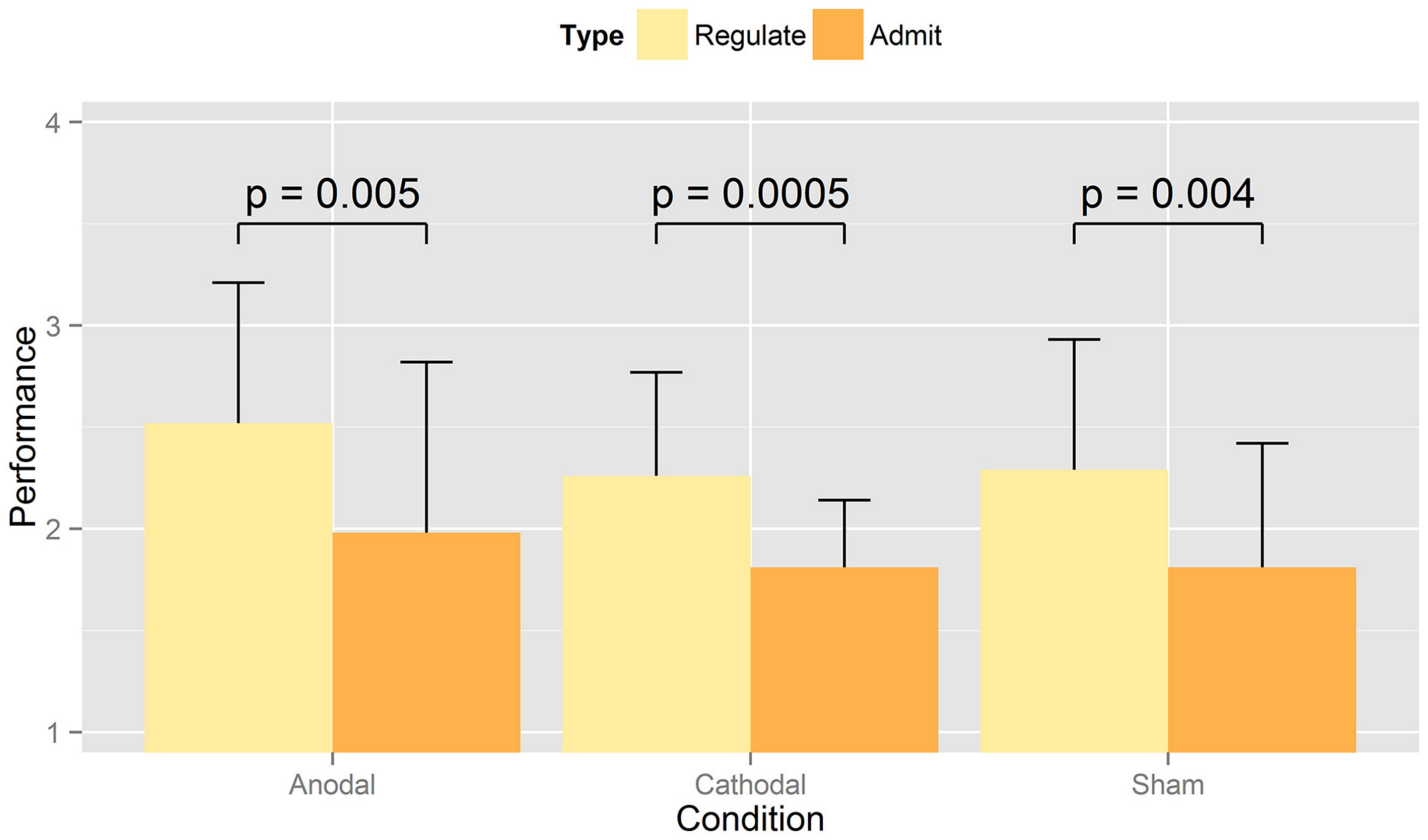
Mean values of performance self-rating. The figure shows the averaged self-ratings indicated by participants when regulating or admitting the desire for presented food for anodal, cathodal, or sham tDCS. Ratings were given on a scale from 1 (“very good”) to 4 (“poorly”). As expected, RM-ANOVA revealed a significant effect between admit and regulate, but not between tDCS conditions (i.e., sham, anodal, cathodal) When comparing the ability to admit vs. regulate, paired *t*-tests showed a significant difference within all three stimulation types.

Participants rated their success in either admitting or regulating their desire for displayed food pictures during the computer task on a scale from 1 (“very good”) to 4 (“poorly”; Tables 5A,B). The strategies that participants used to regulate their food desire are listed in Table 6. RM-ANOVA with the independent variables *condition* (anodal vs. cathodal vs. sham) and *cognitive task* (admit vs. regulate) showed a significant effect of *cognitive task* [*M*_(admit)_ = 1.9, *M*_(regulate)_ = 2.4, *F*_(1, 22)_ = 16.358, *p* = 0.001, *n* = 23], independent from tDCS type or sham (Figure 3). Paired *t*-tests comparing the self-ratings of high to low caloric food pictures in the admit and regulate task separately, disclosed lower ratings for low vs. high caloric in the admit [*M*_(hc)_ = 1.83, *SD* = 0.45; *M*_(lc)_ = 1.67, *SD* = 0.41; *p* < 0.002; *n* = 23] but no difference in the regulate condition [*M*_(hc)_ = 2.23, *SD* = 0.47; *M*_(lc)_ = 2.26, *SD* = 0.54; *p* > 0.6; *n* = 23]. A following RM-ANOVA did not reveal a significant impact of tDCS condition on the self-ratings [*F*_(2, 44)_ = 0.548, *p* = 0.582, *n* = 23]. Self-ratings also indicated higher mean values when participants regulated their desire for sweet compared to salty food [*M*_(sweet)_ = 2.4, *M*_(salty)_ = 2.2, *p* < 0.002, *n* = 23] and vice versa when admitting [*M*_(sweet)_ = 1.7, *M*_(salty)_ = 1.8, *p* < 0.005, *n* = 23], but once again independent of tDCS condition [regulate: *F*_(2, 44)_ = 0.148, *p* = 0.862, *n* = 23; admit: *F*_(2, 44)_ = 0.008, *p* = 0.992, *n* = 23].

**Table 5:** Performance values.

	**Anodal**	**Cathodal**	**Sham**
**A**. Admit			
Admit to high caloric salty food	1.95 (0.57)	1.95 (0.49)	1.89 (0.5)
Admit to high caloric sweet food	1.75 (0.5)	1.72 (0.56)	1.76 (0.59)
Admit to low caloric salty food	1.73 (0.5)	1.82 (0.52)	1.69 (0.5)
Admit to low caloric sweet food	1.57 (0.52)	1.55 (0.45)	1.64 (0.7)
**B**. Regulate			
Regulate high caloric salty food	2.2 (0.58)	2.12 (0.52)	2.21 (0.37)
Regulate high caloric sweet food	2.21 (0.54)	2.45 (0.65)	2.14 (0.55)
Regulate high calroc salty food	2.1 (0.72)	2.21 (0.56)	2.02 (0.45)
Regulate high caloric sweet food	2.47 (0.71)	2.42 (0.67)	2.33 (0.62)

*Displayed are mean self-performance values and standard deviation for each stimulation condition. Participants rated their ability to either admit or regulate their desire for viewed food pictures on a scale from 1 (“very good”) to 4 (“poorly”)*.

**Table 6:** Strategies.

**Regulate**		**Admit**	
**Strategy**	**% (*n*)**	**Strategy**	**% (*n*)**
Attribute negative or disgusting aspect to food item	64 (16)	Imagine taste, feel and consistence	48 (12)
Consider unhealthy aspects and negative consequences	44 (11)	Imagine combination with other foods	36 (9)
Ignore picture, concentrate on something else	28 (7)	Giving in to appetite and feeling of hunger	28 (7)
Talk oneself into, reject	20 (5)	Talk oneself into, forcing	12 (3)
Disliking shown food item	16 (4)	Imagine pleasant surrounding	8 (2)
Ethical aspects (e.g., factory farming)	12 (3)	Consider healthy aspects	4 (1)
Negative association	12 (3)		
Other	8 (2)		

*Overview of used strategies when admitting or regulating the desire for displayed food pictures with frequency of indication in percent and absolute value of usage within cohort (100% is equivalent to n = 25). Participants were permitted to indicate several methods which are listed according to their popularity*.

### Buffet

**Figure 4:**
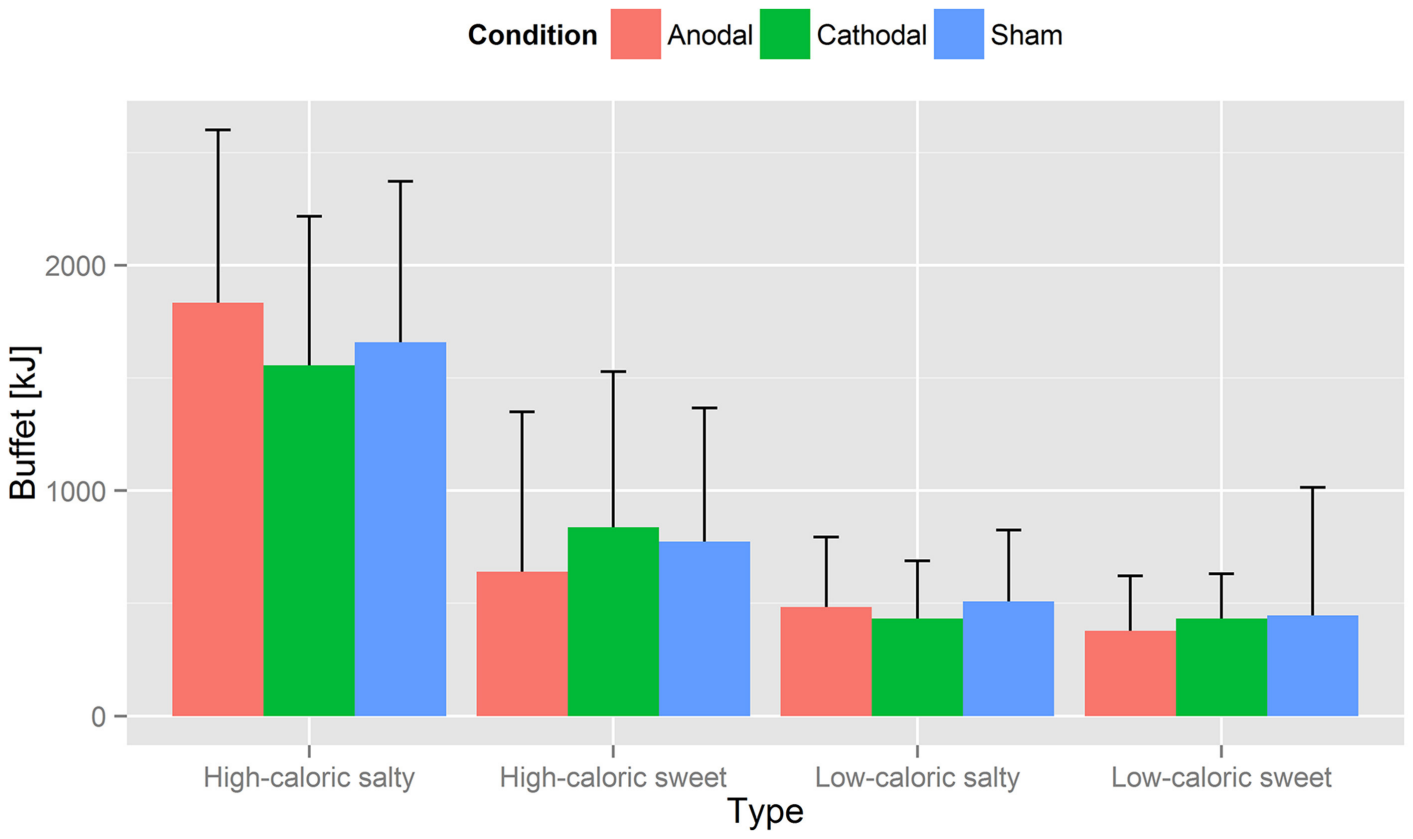
Caloric intake. Displayed is the averaged calorie intake for the four food categories *high caloric salty/sweet* and *low caloric salty/sweet* in kcal, grouped by tDCS type. Values are listed in Table 7. Although participants showed more food consumption after sham stimulation, RM-ANOVA could not suggest an underlying effect of tDCS condition [*F*_(2, 48)_ = 0.16, *p* = 0.853, *n* = 25]. Latter also applied to the subordinate categories *high caloric sweet/salty* and *low caloric sweet/salty*: intake was not reduced significantly after anodal or cathodal tDCS [high caloric salty: *F*_(2, 48)_ = 3.29, *p* = 0.051]; high caloric sweet: *F*_(2, 48)_ = 0.96, *p* = 0.38, *n* = 25; low caloric salty: *F*_(2, 48)_ = 1.17, *p* = 0.318, *n* = 25; low caloric sweet *F*_(2, 48)_ = 0.36, *p* = 0.702, *n* = 25.

Since the experiments lasted from June until November, total caloric intake results were assigned to either a summer (June–August) or fall (September–November) group and compared by unpaired *t*-test. The *p* < 0.01 indicated a season depending eating behavior which valued for a correction to prevent interaction with tDCS induced changes in eating behavior. We therefore compared total caloric intake of each summer and fall group within the control condition (i.e., sham tDCS) and discovered a mean difference of 26% [*M*_(summer)_ = 3708.8 kJ (886 kcal), *M*_(fall)_ = 4656.1 kJ (1112.3 kcal)]. Accordingly, all fall values were subtracted by 26% of their amount. Mean caloric intake, corrected in this manner, is displayed in Table 7, separately for the categories *high caloric sweet/salty* and *low caloric sweet/salty* and for each stimulation condition. Although participants showed more food consumption after sham [*M* = 3386.9 kJ (809.1 kcal)] then cathodal [*M* = 3260.1 kJ (778.8 kcal)] or anodal tDCS [*M* = 3335.8 kJ (796.9 kcal)], RM-ANOVA could not suggest an underlying effect of tDCS condition [*F*_(2, 48)_ = 0.16, *p* = 0.853, *n* = 25]. Latter also applied to the subordinate categories *high caloric sweet/salty* and *low caloric sweet/salty*, where a significantly reduced intake after anodal or cathodal tDCS was not found: high caloric salty [*F*_(2, 48)_ = 3.29, *p* = 0.051], high caloric sweet [*F*_(2, 48)_ = 0.96, *p* = 0.38, *n* = 25], low caloric salty [*F*_(2, 48)_ = 1.17, *p* = 0.318, *n* = 25], low caloric sweet [*F*_(2, 48)_ = 0.36, *p* = 0.702, *n* = 25; Figure 4]. The mean caloric intake of each session [*M*_(1)_ = 3206.1 kJ (765.9 kcal), *M*_(2)_ = 3282.2 kJ (784.1 kcal), *M*_(3)_ = 3468.1 kJ (828.5 kcal)] did not differ significantly over time [*p*_(1 vs. 2)_ = 0.650; *p*_(2 vs. 3)_ = 0.439; *n* = 25] which suggests no verifiable adaption to tDCS. Because no participant exceeded the mean calorie intake plus two standard deviations (~95% threshold) in each tDCS condition dataset, we have no assumption of binge eating behavior within the scope of our experiment. Whereas, the participants' caloric intake did not correlate with their fasting blood sugar levels (Table 2), it did however show a significant correlation with the blood sugar levels after satiation but only under cathodal and anodal tDCS (cathodal: *r* = 0.5, *p* < 0.02; anodal: *r* = 0.6, *p* < 0.001; *n* = 25) not sham (*p* > 0.1, *n* = 25). Taking the VAS scores for tiredness and stress as covariates of caloric intake in a RM-ANOVA showed no significant impact. To additionally assess whether present findings were driven by participants with a BMI > 40 (i.e., morbid obesity), we additionally analyzed the data after their exclusion. This analysis replicated the findings for the full sample suggesting that the observed effects were not driven by BMI outliers. Additionally, we accounted for age as a covariate in RM-ANOVA analyses to eliminate its potential confounding influence (18–43).

**Table 7:** Mean values and standard deviation of caloric intake displayed for the four food categories *high caloric salty/sweet* and *low caloric salty/sweet* in kcal, grouped by tDCS type.

	**High caloric salty**	**High caloric sweet**	**Low caloric salty**	**Low caloric sweet**	**Total**
Anodal	371.9 ± 157.9	200.1 ± 165.1	103.4 ± 61	103.4 ± 47.2	778.8 ± 240
Cathodal	438 ± 183.1	152.9 ± 169.5	115.6 ± 74.2	90.4 ± 58.2	796.9 ± 275.4
Sham	396.1 ± 170.6	184.8 ± 141.8	121.5 ± 75.5	106.6 ± 135.7	809.1 ± 277.9

*Active tDCS (anodal or cathodal) had no significant effect on food consumption (Figure 4)*.

## Discussion

In the present study, we investigated the effect of tDCS over the left DLPFC and right frontal operculum in obese women, firstly on the active reappraisal of visually presented high and low caloric food items and secondly on subsequent food consumption. We hypothesized that the self-reported ability to either regulate or admit the desire for presented food pictures would vary between cathodal and anodal/sham tDCS. Furthermore, we expected that the consumption of palatable food, offered in an appealing buffet, would be down-regulated by previous cathodal tDCS in comparison to anodal/sham stimulation. The present findings could not confirm an effect of tDCS, neither on regulating/admitting the desire for foods, nor on calorie consumption. In all three tDCS conditions, participants rated the ability to admit the desire for the visually presented foods as significantly easier than the reappraisal of the same foods. This was to be expected considering the fasting state of 5 h prior to the experiments.

The DLPFC is believed to represent an essential component of the complex network controlling eating behavior, particularly the cognitive control over presented food cues in everyday life and food reward processing (Hollmann et al., 2012, 2013). The frontal operculum, instead, is believed to primarily host gustatory processes (Rolls et al., 1988; Zatorre et al., 1992; Small et al., 1999), but recent findings also suggest its contribution to higher cognitive processes, such as regulating food desire (Kumar et al., 2016). Numerous studies have started investigating brain areas, mainly implied in the suppression of palatable foods (Schlögl et al., 2016). Their findings highlight an almost disclosed brain circuit involving mesocorticolimbic regions as well as the frontal operculum and the DLPFC (Hollmann et al., 2012; Siep et al., 2012; Kumar et al., 2016). Our present results suggest that endogenous suppression techniques (i.e., reappraisal strategies, see Table 6) associated with one-time extraneous modulation via non-invasive brain stimulation, directed to just two of the circuit's components is not sufficient to modulate food reappraisal abilities or food consumption in obese females. The interpretation of such null findings is generally problematic, since effects may become significant with increasing the sample size or number of tDCS sessions. Nevertheless, as compared to previous studies on tDCS and food choices (Fregni et al., 2008; Goldman et al., 2011; Montenegro et al., 2012; Jauch-Chara et al., 2014; Kekic et al., 2014; Gluck et al., 2015; Ljubisavljevic et al., 2016), we here targeted distinct brain sites derived from recent pilot experiments that were conducted with the same food picture task and hence with the same task demands as in the present study (Kumar et al., 2016). Furthermore, our tDCS study included the largest human obese sample so far. To avoid gender variability as a well-known confounding effect (Horstmann et al., 2011; Mueller K. et al., 2011; Melasch et al., 2016), we focused on the female brain. A major limitation of our study is, however, that menstrual cycle was not inquired. Since, menstrual cycle has effects on food perception and intake (Bryant et al., 2017), it should be considered for future studies investigating the effects of non-invasive stimulation of the female brain on food evaluation and consumption.

In obese individuals, the right prefrontal cortex (PFC) seems to present crucial functional differences when compared to lean individuals, founding the “right brain hypothesis” (Alonso-Alonso and Pascual-Leone, 2007). It suggests that dysregulation, in particular reduced activity in the right PFC, contributes to enhanced probability of overeating, inactivity, reduced capacity of self-reflection on dietary choices and general deficits in decision-making. Also, the left DLPFC was shown to be critically involved in food choices and hence in the control of eating behavior (Gluck et al., 2015), adding evidence to the strong assumption of a prefrontal imbalance in obesity (Carnell et al., 2012; Brooks et al., 2013; Vainik et al., 2013). In our recently published EEG study (Kumar et al., 2016), we also found such a hemispheric imbalance, however, not between both prefrontal cortices, but between the left prefrontal (i.e., DLPFC) and the right frontal cortex (i.e., frontal operculum). Activity in the left DLPFC increased while allowing the desire for food, whereas activity in the right frontal operculum increased with the ability to regulate the desire for food (Kumar et al., 2016). Based on these findings, we here hypothesized that tDCS with its two polarities (i.e., anodal and cathodal) downregulates activity in the left DLPFC, via cathodal stimulation, while simultaneously upregulating activity in the right frontal operculum, via simultaneous anodal stimulation. We expected that this tDCS effect on prefrontal/frontal activity levels strengthens the ability to regulate food desire as well as reduces calorie consumption, which we could not confirm. This could imply that tDCS failed to provoke up- or downregulation of activity in targeted regions (i.e., left DLPFC and right frontal operculum), and possibly also food-related dopamine responses in central reward regions, such as ventral and dorsal striatum as well as nucleus accumbens, connected with the targeted regions. It is therefore questionable whether other regions such as tDCS of left and right DLPFC, as targeted in former studies (Fregni et al., 2008; Goldman et al., 2011; Kekic et al., 2014; Lapenta et al., 2014), would have provoked a feasible alteration of dopaminergic response arising from structures such as striatum or mediofrontal system (Geiger et al., 2008, 2009) and hence initiated indirect dopamine-dependent behavior modification. Due to the three envisaged tDCS conditions (anodal, cathodal, sham) we renounced further expansion with additional potential control brain regions, since we perceived three experiment sessions for each participant as only just acceptable. However, considering the negative results of our study, the possibility of alternating dopaminergic response in regions deep within the brain (e.g., striatum) through non-invasive stimulation of superficially located areas remains questionable. A directly through tDCS mediated interference of dopamine response in the striatum is rather unlikely, since tDCS as well as alternative transcranial magnetic stimulation (TMS) with likewise implemented procedure protocols lack in necessary depth of penetration.

The field of non-invasive brain stimulation experiments is fairly young and current data in the area of eating behavior is few and controversial (Horvath et al., 2015). As Horvath et al. (2015) reported in a meta-analysis of tDCS outcomes, a majority of study designs were lacking in valid control conditions or double-blinding. The only reliable effect of tDCS, that could be shown in this review, was the modulation of motor evoked potentials after tDCS was applied to the motor cortex. The discrepancy in outcomes between studies with however comparable tDCS protocols suggests that there are several influencing variables, such as the thickness and fat proportion of the scalp (Truong et al., 2012), whose impact on brain stimulation are neither fully investigated nor understood. A recently presented trial by György Buzsáki of New York University (NYU) even demonstrated that hardly 10% of alternating current applied via tDCS to cadaver scalps, measured by implanted electrodes, reached brain tissue (Underwood, 2016). This trial is to be anticipated with interest, serves it more doubt on our knowledge of tDCS effects and its applications. Assuming an actual effect of inhibitory or facilitating tDCS to targeted brain regions, it is conceivable that inhibitory stimulation led to suppression of inhibitory GABAergic interneurons and hence excitation of interconnected neural networks. Consequently, behavioral pattern could have been reinforced through inhibitory tDCS, contrary to hypothesized suppression. Since, we could not evince neither facilitation nor suppression of behavioral patterns under inhibitory tDCS concerning the modulation of food desire as well as actual consumption, inhibition of inhibitory GABAergic interneurons in the context of our work seems unlikely.

Although, food-craving as an addiction-like behavior has been shown to decrease after tDCS (Fregni et al., 2008; Goldman et al., 2011; Kekic et al., 2014; Ljubisavljevic et al., 2016), tDCS effects on food choices, especially with respect to palatable but unhealthy food, in hungry obese females or males remain largely unexplored. Most studies were restricted to participants within a lean BMI range (i.e., BMI < 30; Fregni et al., 2008; Goldman et al., 2011; Jauch-Chara et al., 2014; Kekic et al., 2014). To the best of our knowledge, Gluck et al. (2015) has published the only study so far showing that the repetitive application of anodal prefrontal tDCS to the left DLPFC decreases caloric intake in a small cohort consisting of nine obese males and female, whereas Ljubisavljevic et al. (2016) suggested reduced food craving after anodal stimulation of the right DLPFC in a cohort of normal as well as obese young adults. In our study, we used a single session tDCS because of its well-described effects on food-craving (Jansen et al., 2013; Kekic et al., 2014; Lapenta et al., 2014). Our findings however suggest that a single tDCS session of 20 min is not sufficient to modulate reappraisal strategies as well as calorie intake. Another striking difference to the study by Gluck et al. (2015) is, that we applied tDCS during the reappraisal task and not prior to the task. We decided for this online tDCS design, since Nitsche et al. previously reported that tDCS effects occur already within minutes after tDCS initiation (Nitsche and Paulus, 2000, 2001). Thus, different tasks in combination with different online tDCS protocols may have different immediate influences on participants' actual brain function and behavior. Future methodological tDCS studies are necessary to compare tDCS in combination with online or offline tasks in order to assess their specific advantages or disadvantages.

The laboratory settings of previous (Gluck et al., 2015; Ljubisavljevic et al., 2016) and the present study, just as much as the placing of our standardized buffet in an investigation room, stresses the distance to habitual eating behavior in a familiar social environment. Heightened awareness of observation under lab conditions, for instance, was shown to cause obese females to reduce their calorie consumption (Robinson et al., 2016), suggesting social modeling as an important influencing factor on eating behavior. Cruwys et al. (2015) reviewed several studies published between 1974 and 2014 and found that social modeling of eating seems at least to be partially mediated through behavioral mimicry, which occurs without conscious awareness. Since participants in our study were alone at the buffet, there was no “ideal model” they may have desired to affiliate with. This makes social modeling rather unlikely to account for potential reluctance in eating. Each participant was furthermore investigated three times (i.e., sham, anodal, cathodal tDCS) in random order under similar conditions, canceling out possible influences of social modeling, if tDCS conditions are statistically compared against each other.

Another potentially influencing factor on the present findings is the heterogeneity of obesity phenotypes together with heterogeneous eating behavior traits, specifically with respect to impulsivity and disinhibition. To assess the influence of those traits on our results, we investigated the relation between grade of obesity and impulsiveness as well as temper control through correlation analysis of BMI and the BIS-15 as well as AT questionnaires, which revealed no verifiable coherence. The BDI was used for initial screening of indicators for depression and participants scoring more than 15 points were excluded from the study. However, no further diagnostic tool was instated, which also applies for anxiety disorders that could be associated to binge eating behavior (Mitchell et al., 1999; Ostrovsky et al., 2013). Another aspect of the present study design, that may have accounted for data heterogeneity and hence the lack of tDCS effects, is the strategy participants used to regulate their food desire. As in previous studies (Hollmann et al., 2012; Kumar et al., 2016), we specifically allowed participants to freely choose the best strategies. We hypothesized that this approach supports optimal individual food regulation abilities. However, Siep et al. (2012) found that short-term suppression of food desire is more successful in inhibiting corresponding brain activation then cognitive restraint, hence thinking of long-term consequences. Women of our study however used either short-term or long-term consequences as strategies for regulating their food desire (Table 6).

A number of previous studies investigated the effect of non-invasive brain stimulation linked with cognitive restraint training and even physical activity on food choices, eating behavior, or calorie consumption. Controversial findings due to diverse study designs, sample sizes, and stimulation response in individuals called for supplemental research (Barth et al., 2011; Horvath et al., 2015). Our fully randomized, within-subject, placebo-controlled, and double-blinded study does not support the notion of tDCS as a promising method to improve the regulation of food desire or food consumption in obese women. However, our results are in disagreement with previous pilot trials by Gluck et al. who used repetitive application of tDCS (Gluck et al., 2015). This discrepancy between the present and previous findings demands further studies combining a comparable study design as in the present study with repetitive tDCS instead of just one single session.

## Ethics statement

This study was carried out in accordance with the recommendations of the ethics committee of the medical faculty of the University of Leipzig with written informed consent from all subjects. All subjects gave written informed consent in accordance with the Declaration of Helsinki. The protocol was approved by the ethics committee of the medical faculty of the University of Leipzig.

## Author contributions

Conception and design of study: FG, CB, SK, JM, BP. Acquisition of data: FG, CB. Analysis and/or interpretation of data: FG, CB, MR, JM, BP. Drafting the manuscript: FG, BP. Approval of the version of the manuscript to be published: FG, CB, SK, MR, JM, BP.

### Conflict of interest statement

The authors declare that the research was conducted in the absence of any commercial or financial relationships that could be construed as a potential conflict of interest.
